# Ultrasound criteria for transmural healing and response in Crohn’s disease: a systematic review of definitions and thresholds

**DOI:** 10.1093/ibd/izag052

**Published:** 2026-06-01

**Authors:** Joëlle St-Pierre, Maxime Delisle, Yusuke Miyatani, Katherine Falloon, Kenneth Ernest-Suarez, Baldeep Pabla, Hien Huynh, Brooke Maracle, Janice Y Kung, Noa Cleveland, David T Rubin, Michael Dolinger, Kerri Novak, Oriana Damas, Gil Y Melmed, Cathy Lu, Amelia Kellar

**Affiliations:** IBD Unit, Division of Gastroenterology and Hepatology, Department of Medicine, University of Calgary, Calgary, AB, Canada; Centre Hospitalier Universitaire de Sherbrooke, Sherbrooke, QC, Canada; Department of Medicine, John A. Burns School of Medicine, University of Hawai’i, Honolulu, HI, United States; Gastroenterology, Hepatology, and Nutrition, Digestive Disease and Surgery Institute, Cleveland Clinic Foundation, Cleveland, OH, United States; Faculty of Medicine, University of Costa Rica, San José, Costa Rica; Vanderbilt Health, Vanderbilt University, Nashville, TN, United States; Department of Pediatrics, Faculty of Medicine & Dentistry, University of Alberta, Edmonton, AB, Canada; IBD Unit, Division of Gastroenterology and Hepatology, Department of Medicine, University of Calgary, Calgary, AB, Canada; Geoffrey & Robyn Sperber Health Sciences Library, University of Alberta, Edmonton, AB, Canada; University of Chicago Medicine Inflammatory Bowel Disease Center, Chicago, IL, United States; University of Chicago Medicine Inflammatory Bowel Disease Center, Chicago, IL, United States; Division of Pediatric Gastroenterology, Hepatology, and Nutrition, NYU Grossman School of Medicine, New York, NY, United States; IBD Unit, Division of Gastroenterology and Hepatology, Department of Medicine, University of Calgary, Calgary, AB, Canada; Division of Digestive Health and Liver Diseases, University of Miami, Miami, FL, United States; Cedars-Sinai Medical Center, Los Angeles, CA, United States; IBD Unit, Division of Gastroenterology and Hepatology, Department of Medicine, University of Calgary, Calgary, AB, Canada; University of Chicago Medicine Inflammatory Bowel Disease Center, Chicago, IL, United States; Department of Pediatrics, Section of Gastroenterology, Hepatology and Nutrition, University of Chicago, Chicago, IL, United States

**Keywords:** intestinal ultrasound, Crohn’s disease, transmural healing, transmural remission

## Abstract

**Background:**

Transmural healing (TMH) indicates resolution of inflammation in all bowel wall layers and is an emerging therapeutic target in Crohn’s disease (CD). Standardized sonographic criteria for TMH and early improvement, termed Transmural Response (TMR), have not been established. This systematic review synthesizes published definitions to provide an up-to-date overview of the current evidence base for intestinal ultrasound (IUS)-based assessment in CD.

**Methods:**

This systematic review was conducted in accordance with the Preferred Reporting Items for Systematic Reviews and Meta-Analyses guidelines. Comprehensive searches of databases identified full-text articles that pre-specified TMH, TMR or normal/abnormal bowel on trans-abdominal IUS in pediatric or adult participants with CD. Definitions were summarized descriptively.

**Results:**

Eighty-three full-text studies (8033 patients) met eligibility criteria; 39 (47%) defined TMH and 22 (27%) defined TMR. TMH definitions most often included bowel-wall thickness (BWT) ≤ 3mm (31/39, 79%), absent or minimal Doppler flow (25/39, 64%), and preserved bowel wall stratification (10/39, 26%). All TMR definitions required BWT reduction, but thresholds varied (absolute ≥ 1 mm or relative ≥ 25% in 16/22, 73%). Nine studies (9/22, 41%) also required Doppler flow improvement and 4/22 (18%) included additional criteria. Pediatric-specific criteria were reported in 2 TMH and one TMR studies, extrapolating from adult BWT values. Heterogeneity precluded quantitative pooling.

**Conclusions:**

Standardized IUS definitions of TMH and TMR in CD are lacking. Consistent, validated criteria are essential to enable reproducible ultrasound endpoints, support treat-to-target strategies, and facilitate incorporation of IUS into CD clinical trials and routine care.

Key Messages

**What is already known?** Transmural healing assessed by intestinal ultrasound is an emerging therapeutic target in Crohn’s disease, but definitions of healing and response vary widely across studies.
**What is new here?** This systematic review synthesizes all published sonographic definitions of transmural healing and transmural response, demonstrating that bowel wall thickness and color Doppler flow are the most consistent parameters included in definitions, while ancillary criteria are inconsistently applied.
**How can this study help patient care?** Standardized ultrasound definitions will enable reproducible assessment, improve treat-to-target implementation, and support the inclusion of sonographic endpoints in future Crohn’s disease clinical trials.

## Introduction

Management of Crohn’s disease (CD) has progressively shifted from symptom control to a treat-to-target paradigm, emphasizing the objective resolution of inflammation to better predict long-term outcomes. The STRIDE II consensus statement proposes deep, durable control of disease activity as a therapeutic goal in daily clinical practice and trials.^1^ Robust evidence was lacking during the development of STRIDE II to include cross-sectional imaging as a formal treatment target. Nevertheless, the panel specifically noted radiologic (including sonographic) response as a promising future target once supportive data accumulate. Within this framework, mounting evidence now demonstrates that complete resolution of transmural inflammation, defined as transmural healing (TMH), confers even greater prognostic benefit than endoscopic mucosal healing alone.^2^ In a multicenter cohort of patients treated with biologics, those that achieved TMH experienced markedly lower rates of relapse, hospitalization, and surgery after one year compared with those who achieved mucosal healing or no healing.^3^ As complete bowel wall healing is a longitudinal target, transmural response (TMR) which includes partial interval improvement, has emerged as an attractive early-intervention surrogate.

Intestinal ultrasound (IUS) is uniquely positioned to assess TMH. In the prospective, multicenter TRansabdominal Ultrasonography of the bowel in Subjects with IBD To monitor disease activity (TRUST) study, serial IUS not only accurately paralleled clinical and biochemical activity, but also captured early transmural changes, while remaining radiation-free, inexpensive, and highly acceptable to patients.^4^ Subsequent real-world cohorts and expert society statements reinforce IUS as a frontline modality for tight-control CD management and highlight its expanding use in clinical trials.^5–10^

Despite the widespread use of IUS, standardized definitions for TMH and TMR are lacking. A 2022 systematic review and expert IUS consensus identified wide variation in normalized values for bowel-wall thickness (BWT), assessment of vascularity and ancillary criteria used to define IUS remission or response, underscoring the need for harmonization.^11^ A more recent comprehensive review of 51 studies, published in 2025, confirms persistent heterogeneity, particularly with regards to the magnitude of BWT change required for TMR and the composite parameters incorporated into TMH definitions.^12^ This inconsistency hampers cross-study comparison, meta-analysis and regulatory acceptance of IUS-based trial endpoints.

Accordingly, the present systematic review aims to (1) catalogue all a priori sonographic definitions of TMH and TMR in CD, (2) dissect their constituent parameters and cut-offs, (3) evaluate supporting validation data, and (4) propose a pragmatic, hierarchically tiered framework to guide future consensus and prospective validation. By synthesizing the rapidly expanding literature, we seek to lay the evidentiary foundation necessary to standardize IUS therapeutic targets and accelerate treat-to-target implementation for patients living with CD.

## Methods

### Search strategy

A comprehensive literature search was conducted across multiple electronic databases, including Ovid MEDLINE, Ovid Embase, CINAHL, Web of Science Core Collection, Cochrane Library (via Wiley), and ClinicalTrials.gov. The search strategy was designed in collaboration with an experienced health sciences librarian to ensure comprehensiveness. Search terms combined relevant keywords and controlled vocabulary (eg, MeSH headings) including TMH, TMR, IUS, CD, and bowel ultrasound. Boolean operators and database-specific syntax were applied to refine the search, which was limited to studies published in English. Studies published up to February 2025 were eligible for inclusion. Search results were uploaded into Covidence for deduplication and screening. Grey literature, including conference abstracts from gastroenterology and IBD-focused meetings, was also reviewed. The protocol is registered with PROSPERO (CRD42024519934). The reporting of this systematic review was guided by the standards of the Preferred Reporting Items for Systematic Review and Meta-Analysis (PRISMA) Statement.

### Inclusion/exclusion criteria

Titles and abstracts were independently screened in duplicate: each citation was assessed by 2 members of the reviewer team against the predefined inclusion and exclusion criteria. Studies were included if they employed interventional designs (randomized or non-randomized), observational designs (prospective, retrospective, or case-control), or case series including more than 10 patients. Eligible studies focused on adults and/or pediatric patients with CD and evaluated TMH or TMR using transabdominal IUS. Studies that reported only post hoc or ROC-derived cut-offs without pre-specified sonographic definitions were excluded from pooled analyses. Studies focusing on perianal or endoanal ultrasound were excluded. Systematic reviews or meta-analyses were excluded to avoid duplicating findings.

### Outcomes and data extraction

The primary outcomes were the definitions of TMH and TMR as assessed by transabdominal IUS. Studies that reported only post hoc or ROC-derived cut-offs, without a pre-specified sonographic definition, were catalogued descriptively, but excluded from the pooled frequency analyses of predefined criteria. Definitions were evaluated for consistency across studies, focusing on parameters such as bowel wall thickness (BWT), hyperemia, bowel wall stratification, and the presence of complications such as strictures and/or fistulas. Secondary outcomes included correlations between IUS-based definitions and clinical, biochemical, and additional cross-sectional imaging modality metrics. These included clinical indices such as the simple endoscopic score for CD (SES-CD) and the Harvey-Bradshaw index (HBI),^13^^,^^14^ biochemical markers (eg, c-reactive protein [CRP], fecal calprotectin), imaging findings, and therapeutic interventions, along with timelines for achieving TMH and TMR.

Data extraction was performed by one reviewer using a standardized form to capture study characteristics (design, setting, sample size, and follow-up duration), participant demographics, IUS parameters, and patient outcomes. Information was then verified by a second reviewer to minimize errors. Specific criteria for remission and response, clinical and biochemical correlations, and details of therapeutic interventions (eg, medication and dosing regimens) were recorded. Data were summarized descriptively, and no data conversions or imputations were performed. When quantitative values were incomplete, results were reported narratively using information as presented in the original publication.

### Risk of bias assessment

The overall certainty of evidence was not formally graded using the GRADE framework, as quantitative synthesis was not performed. Instead, the risk of bias for included studies was assessed using the Joanna Briggs Institute (JBI) Critical Appraisal Tool, which provides a structured approach for evaluating methodological quality across different study designs.^15^^,^^16^

## Results

### Study characteristics

**Figure 1:**
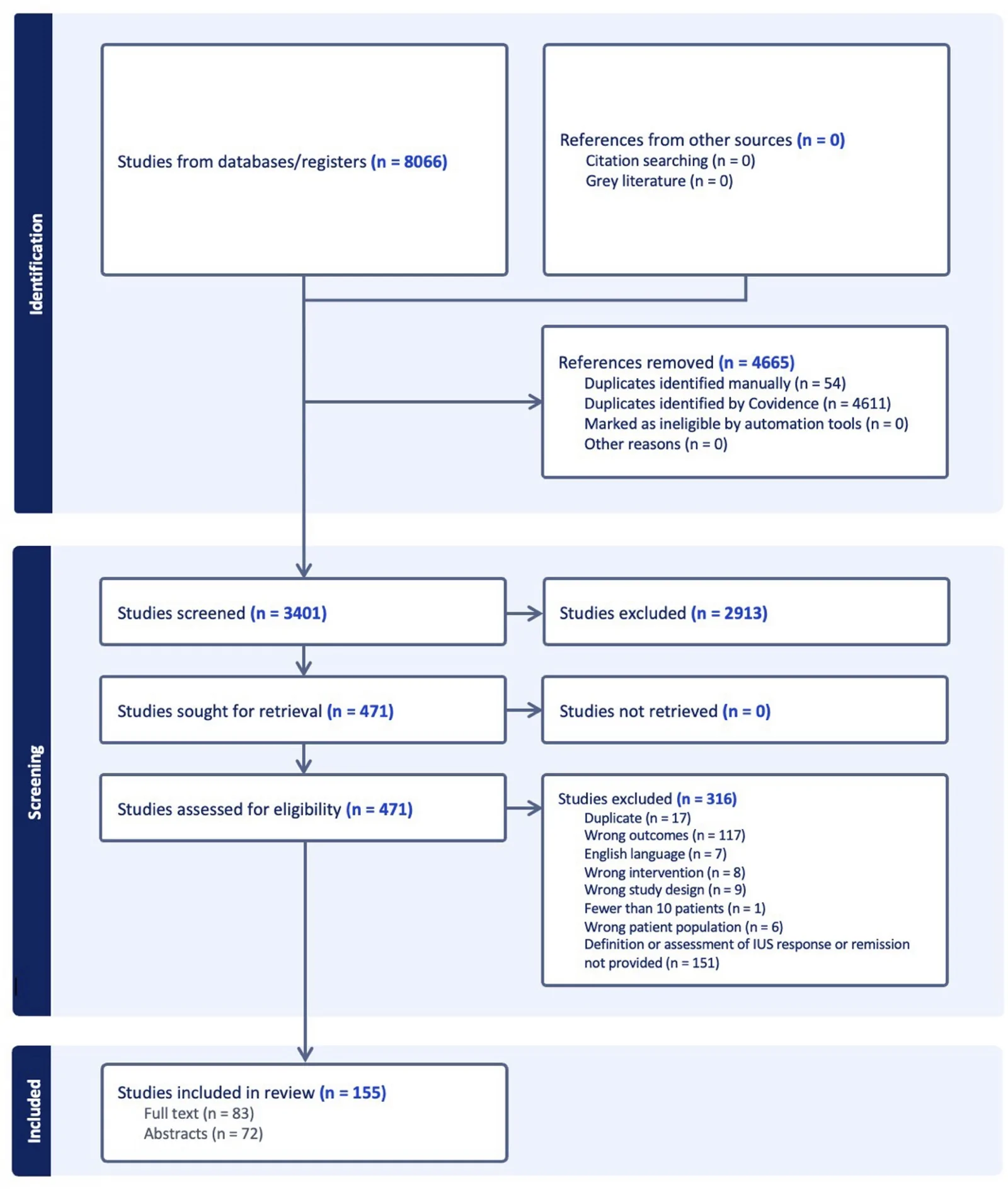
PRISMA 2020 study-selection flow diagram. The diagram traces the identification, screening, eligibility assessment and inclusion of reports that proposed or applied intestinal-ultrasound definitions of TMH, TMR, or normal/abnormal bowel in CD. After duplicate removal, titles and abstracts were screened, full-text articles were reviewed for eligibility with reasons for exclusion documented, and the final synthesis comprised 83 peer-reviewed studies and 66 conference abstracts. Boxes display the corresponding record counts at each stage.

A total of 83 studies met inclusion criteria (Figure 1), with publication dates ranging from 1992 to 2025 (median 2021). The majority of studies were cohort-based (62/83, 74.7%), followed by cross-sectional design (19/83, 22.9%), one randomized controlled trial (1/83, 1.2%), and one case-control study (1/83, 1.2%). Collectively, the studies enrolled 8033 participants (median 77, [43.5-115.5]). Special populations were relatively uncommon: 9 studies (10.8%) enrolled pediatric patients (total of 190 participants with CD) and one study focused on pregnant individuals (22 participants with CD). The 83 eligible studies were published from 18 different countries spanning 5 continents: Europe accounted for the majority of studies (51/83, 61.4%), followed by Asia (18/83, 21.7%), North America (7/83, 8.4%), Oceania (5/83, 6.0%), and Africa (1/83, 1.2%). One additional study was a multinational collaboration from Europe (1/83, 1.2%). Risk of bias was assessed using the JBI critical appraisal tools according to study design (Table S1). Overall methodological quality was moderate across studies. Most observational studies clearly defined inclusion criteria and applied standardized ultrasound protocols, but few provided details on blinding of image interpretation or assessor training. Sample size justification and handling of missing data were rarely reported. Interobserver reliability was assessed in only a minority of studies.

### Definitions of transmural healing

Thirty-nine full-text manuscripts provided an a priori ultrasound definition of TMH (Table 1). The reported criteria converged into 3 progressively stringent categories. The least complex group relied solely on BWT, almost invariably adopting a threshold of ≤ 3 mm for any intestinal segment. When segment-specific thresholds were reported, some studies allowed values up to 4 mm in the rectum or colon. These BWT-only parameters accounted for 23% (9/39) of definitions.^3^^,^^17–24^ A second, more common pattern (23%; 9/39) combined BWT ≤ 3 mm and an absent or near-absent color Doppler signal (CDS), or as Limberg modified Limberg grade 0-1.^25-33^ The most granular pattern was reported by 49% (19/39) of studies, which included one or more structural or peri-enteric complication criteria (ie preserved bowel wall stratification, absence of mesenteric fat wrapping, and/or absence of fistulas or strictures). Of these multicomponent definitions in 19 studies, 15 also retained a color Doppler signal requirement. Two studies replaced single-parameter rules with the Bowel Ultrasound Segmental Score (BUSS) < 3.52, which is a composite index integrating BWT, CDS, mesenteric fat, and lymphadenopathy.^34^^,^^35^ Helwig et al. (2022) listed 3 definitions separately, described as simplified (normalization of bowel wall thickness and color Doppler signal), extended (normalization of bowel wall thickness, color Doppler, and at least 2 additional parameters: preserved stratification or absence of fibro-fatty proliferation), and complete (normalization of all 4 parameters: bowel wall thickness, color Doppler, stratification, and fibro-fatty proliferation) variants that mirror the gradations above.^36^

**Table 1:** Grouped definitions of TMH in patients with CD.

Elements included in the definitions	Definition	Studies
**BWT only**	BWT ≤ 3 mm	Castiglione et al. 2013; Castiglione et al. 2017; Orlando et al. 2018^a^; Castiglione et al. 2019 ; Miranda et al. 2021; Castiglione et al. 2022 ; Chen et al. 2022 ; Wu et al. 2022
	BWT ≤ 4 mm	Marin et al. 2021
**BWT and hyperemia**	BWT ≤ 3 mm and absence of hyperemia	Suarez Ferrer et al. 2021; Spencer et al. 2023 ; Smith et al. 2022 ; Vaughan et al. 2023 ; Guo et al. 2025^b^
	BWT ≤ 3 mm for TI/colon and ≤ 4 mm for rectum, and absence of hyperemia	Saevik et al. 2022
	BWT ≤ 3 mm and CDS ≤ 1	Paredes et al. 2019; Han et al. 2022
	BWT ≤ 3 mm, CDS ≤ 1, and parietal enhancement increase of < 46% (measured by CEUS)	Moreno et al. 2014
	TI BWT ≤ 3 mm and CDS < 2	Ukashi et al. 2024
**BWT, hyperemia and complications**	BWT ≤ 3 mm for TI and ≤ 4 mm for colon, and absence of complications	Onali et al. 2022; Wu et al. 2025
	BWT ≤ 3 mm, absence of hyperemia, and absence of complications	Paredes et al. 2010; Ripolles et al. 2016 ; Lorente et al. 2024
	BWT ≤ 3 mm for TI and ≤ 4 mm for colon, absence of hyperemia, and absence of complications	Zorzi et al. 2020; Calabrese et al. 2022
**BWT, hyperemia, mesenteric fat wrapping and echostratification**	BWT ≤ 3 mm, absence of hyperemia, preserved echostratification, and absence of mesenteric fat wrapping	Vaughan et al. 2022; Kucharzik et al. 2023
	BWT ≤ 3 mm, CDS ≤ 1, preserved echostratification, and absence of mesenteric fat wrapping	Cheng et al. 2023; Maconi et al. 2024; Huang et al. 2024
**BWT, hyperemia, mesenteric fat wrapping, echostratification and complications**	BWT ≤ 2 mm for TI and ≤ 3 mm for colon, absence of hyperemia, preserved echostratification, absence of mesenteric fat wrapping, and absence of complications	De Voogd et al. 2022
	BWT ≤ 3 mm, absence of hyperemia, preserved echostratification, absence of mesenteric fat wrapping, and absence of complications	Civitelli et al. 2016^b^; Chen et al. 2019
	BWT ≤ 3 mm, CDS ≤ 1, preserved echostratification, absence of mesenteric fat wrapping, and absence of complications	Ma et al. 2021
**Composite score**	BUSS score < 3.52	Allocca et al. 2022; Allocca et al. 2024
**Other**	1. Simplified TMH (normalized BWT and/or ≥ 25 % reduction plus absent color Doppler) 2. Extended TMH (normalized BWT with ≥ 2 of the following: absent Doppler, preserved stratification, no fibro-fatty proliferation), and 3. Complete TMH (normalized BWT with all 3 adjunct parameters)	Helwig et al. 2022

Abbreviations: BUSS, bowel ultrasound segmental score; BWT, bowel wall thickness; CDS, color Doppler signal; SB, small bowel; TI, terminal ileum.

a Specific to ileal BWT.

b Pediatric population.

Across all studies BWT ≤ 3 mm was the dominant criteria, present in 79% (31/39) of definitions. Complete or near-complete absence of color Doppler flow was featured in 64% (25/39), and preserved stratification was required in 26% (10/39). Reference to mesenteric fat wrapping or transmural complications appeared less often, in 21% (8/39) of studies.

Pediatric studies were limited. Two manuscripts (5%; 2/39) focused exclusively on children.^32^^,^^37^ Both extrapolated from adult normalized values, including a BWT ≤ 3 mm cut-off without age-specific adjustment, and required the absence of color Doppler signal. Guo et al. (2025) also included preserved echostratification, absence of mesenteric fat wrapping and absence of complications to their definition of TMH.^32^

### Definitions of transmural response

Twenty-two full-text manuscripts provided definitions of TMR (Table 2). Across all studies, some degree of BWT change was universal, explicit color Doppler improvement was featured in 9/22 studies (41%), and additional structural or complication criteria were included in 4/22 (18%) of studies. Reported criteria again differentiated into progressively complex patterns. Eight studies (36%) relied exclusively on BWT, either defined as any reduction without a specified cut-off,^38^^,^^39^ a set decrease by 1 or 2 mm,^40^^,^^41^ a percentage reduction from baseline,^42^^,^^43^ or a mix of both.^32^^,^^36^^,^^44^^,^^45^ A second pattern, reported in 6 manuscripts (27%), paired BWT improvement with a concomitant decrease in mural vascularity, most commonly a ≥ 1-grade decrease to Limberg, modified Limberg or CDS.^25^^,^^27^^,^^28^^,^^31^^,^^46^^,^^47^ Three studies (14%) added further structural or inflammatory parameters such as resolution of transmural complications or shortening of inflamed-segment length in addition to BWT and color Doppler criteria.^6^^,^^8^^,^^29^^,^^48^ The remaining 2 studies (9%) replaced individual parameters with validated composite scores, defining response as either a ≥ 1.2-point reduction in BUSS^35^ or a ≥ 25% reduction and/or normalization of the international bowel ultrasound segmental activity score (IBUS-SAS).^49^

**Table 2:** Definitions of TMR in patients with CD.

Elements included in the definitions	Definition	Studies
**BWT only**	Reduction in BWT (no cut-off)	Onali 2022; Wu 2025
	↓ in BWT of ≥ 25%	Kucharzik 2023; Maconi 2024
	↓ in BWT of ≥ 1 mm	Hoffmann 2022
	↓ in BWT of ≥ 2 mm	Ma 2024
	↓ in BWT of ≥ 2mm or ≥ 20%	Maconi 2001
	↓ in BWT of ≥ 25% or a normalization of BWT	Helwig 2022
	↓ in BWT of ≥ 25% or ≥ 2 mm	Guo 2025^a^ Brodersen 2024
**BWT and hyperemia**	↓ in BWT of ≥ 0.5 mm -and- ↓ in CDS grade ≥ 1	Paredes 2010; Smith 2022
	↓ in BWT of ≥ 1 mm -and- ↓ in CDS grade ≥ 1	Han 2022
	↓ in BWT of ≥ 2 mm -and- ↓ in CDS grade ≥ 1	Paredes 2019
	↓ in BWT of ≥ 25% or a normalization of BWT -and- ↓ in CDS grade ≥ 1 (but not from grade 1 to 0)	Ripolles 2008
	↓ in BWT of ≥ 25% -or- ↓ in BWT of ≥ 2 mm -or- ↓ in BWT of ≥ 1 mm + 1 point decrease in mLimberg score	Spencer 2023
**BWT, hyperemia, complications**	↓ in BWT of ≥ 2 mm -and- one-grade decrease in CDS grade -and- ↓ of ≥ 20% of mural enhancement and/or disappearance of transmural complications or stenosis	Ripolles 2016
	↓ in BWT to < 4.5 mm and absence of hyperemia or other inflammatory data but with no normalization	Suarez Ferrer 2021
	↓ in BWT of > 1 mm or normalization of BWT (<3 mm for SB, < 4 mm for colon) -and- ↓ length of disease -and- Limberg score improvement -and- no worsening of the other disease parameters of active inflammation or fistulizing disease.	Calabrese 2022
	↓ in BWT of > 1 mm or normalization of BWT (<3 mm for SB, < 4 mm for colon) -and - ↓ length of disease -and- no worsening of the other disease parameters of active inflammation or fistulizing disease	Zorzi 2020
**Composite scores**	↓ in TI-BWT of >= 25% or IBUS-SAS	Ukashi 2024
	↓ 1.2 points in BUSS score	Allocca 2024

Abbreviations: BUSS, bowel ultrasound score; BWT, bowel wall thickness; CDS, color Doppler signal; IBUS-SAS, international bowel ultrasound segmental activity score.

a Pediatric population.

Pediatric-specific evidence was again limited: only one manuscript assessed TMR exclusively in children.^32^ This study applied the standard adult BWT-only criteria (≥25% reduction or ≥ 2mm) and did not introduce pediatric adjustments for color Doppler or ancillary parameters.

### Definitions of abnormal and normal bowel by IUS

Across the 52 publications that supplied an a priori ultrasound definition of an “abnormal” or “normal” bowel wall without explicitly addressing TMH or TMR (Table 3), 3 hierarchical patterns emerged. First, 38 studies (73%) relied solely on BWT. Reported thresholds varied, ranging from > 1.5 mm in the terminal ileum and > 2.0 mm in the colon^50^ to > 4 mm applied to any segment.^45^^,^^51–55^ The most common cut-off for normal BWT was > 3 mm, which was used in approximately half of definitions (26/52) and typically applied uniformly to both small and large bowel. Four studies provided different thresholds for small bowel and large bowel.^4^^,^^50^^,^^56^^,^^57^

**Table 3:** Definitions of abnormal bowel on IUS in CD.

Elements included in the definitions	Definition	Studies
**BWT only**	BWT > 1.5 mm in TI and > 2.0 mm in large bowel	Haber et al. 2002
	BWT > 2.0 mm in TI and > 3.0 mm in large bowel	Kucharzik et al. 2017
	BWT > 3 mm or ≥ 3 mm	Flanagan et al. 2020; Castiglione et al. 2022; Castiglione et al. 2004; Rispo et al. 2006; Calabrese et al. 2009; Calabrese et al. 2009^a^; Cammarota et al. 2013^a^; Castiglione et al. 2017; Castiglione et al. 2019; Furfaro et al. 2023^a^^,^^b^; De Cristofaro et al. 2023; Wang et al. 2024; Wu et al. 2022; Zhou et al. 2023; Broderson et al. 2024; Wilkens et al. 2018; Wu et al. 2022; Goertz et al. 2021; Horjus Talabur Horje et al. 2015; Paredes et al. 2013; Moreno et al. 2014; Martinez et al. 2019^a^; Onali et al. 2010; Dell’Era et al. 2023^c^; Dolinger et al. 2021^c^; Khan et al. 2024^c^
	BWT > 2.5 mm in small bowel and > 3.5 mm in large bowel	El-Nakeep et al. 2024
	BWT > 3.5 mm	Na et al. 2017; Celikyay et al. 2021
	BWT > 4 mm	Maconi et al. 2001^a^; Parente et al. 2004; Hata et al. 1992; Liu et al. 2015; Mayer et al. 2000; Marin et al. 2021
	BWT ≥ 3 mm in small bowel and ≥ 4 mm in large bowel	Nasuno et al. 2023
**BWT and hyperemia**	BWT ≥ 3 mm or presence of hyperemia	Yzet et al. 2024; Macedo et al. 2022; Gu et al. 2024
	BWT ≥ 3 mm and presence of hyperemia	Pascu et al. 2004; Schulberg et al. 2022; Aomatsu et al. 2011^b^^,^^c^; Ruess et al. 2000^c^
	BWT ≥ 3 mm or mLimberg > 1	Hoffman et al. 2022; Ripolles et al. 2008
	BWT ≥ 3 mm or CDS > 1	Martinez et al. 2009
**BWT, hyperemia and mesenteric fat wrapping**	BWT > 4 mm, presence of CDS, and presence of mesenteric fat wrapping	Medellin-Kowalewski et al. 2016
**BWT, hyperemia, mesenteric fat wrapping, and other ancillary findings**	BWT ≥ 3 mm and at least one ancillary finding (loss of wall stratification, increased Doppler signal, fat wrapping, loss of haustral marking, and complications such as inflammatory infiltrates, abscess, fistula, or stenosis)	You et al. 2023
	BWT ≥ 2 mm in TI or ≥ 3 mm for the large bowel, and at least one ancillary IUS abnormality (CDS grade ≥ 2 hyperemia, loss of wall stratification, loss of haustration, or mesenteric fat wrapping)	De Voogd et al. 2022 (2847 pregnancy)
	BWT ≥ 3 mm or any ancillary inflammatory sign was present (Limberg grade 3-4 hyperemia, loss of wall stratification, mesenteric inflammatory fat, loss of haustration, absent terminal-ileal motility, or mesenteric lymph-nodes ≥ 5 mm)	De Voogd et al. 2022
**Ultrasound score**	Simple U.S. score > 5.5	Paredes et al. 2022
	SPAUSS > 7	Kellar et al. 2019^c^

a Indicates definitions specific to post-surgical bowel wall.

b Specific to ileal segment.

Simple U.S. score: Simple Ultrasound score = BWT (in mm) + color-Doppler grade.

SPAUSS = BWT points + hyperemia points + mesenteric-fat points (ranges from 0 to 14).

c Pediatric population.

A second group of 9 studies (19%) combined BWT with hyperemia. Some required both features (eg, “BWT ≥ 3 mm and hyperemia”)^58^^,^^59^ whereas others accepted either abnormality in isolation (eg, “BWT ≥ 3 mm or hyperemia”).^60–62^ Whenever color Doppler flow was graded, a CDS score > 1 was the usual threshold.^40^^,^^46^^,^^63^

Four studies (8%) adopted multicomponent criteria, incorporating ancillary signs such as mesenteric fat proliferation, loss of bowel haustration or bowel wall stratification, or transmural complications.^64–67^ Finally, 2 studies used a composite activity index. One used the Simple US score, which is composed of BWT and CDS, with a threshold of > 5.5^68^ and the second using the Simple Pediatric Activity Ultrasound Score (SPAUSS), which integrates BWT, color Doppler signal, and presence or absence of mesenteric fat into a single metric and using a threshold of > 7.^69^

Six pediatric series met the eligibility criteria.^69–74^ One study proposed a dedicated composite index.^69^ The remaining 5 manuscripts applied adult-derived BWT and color Doppler thresholds. Dolinger et al. (2021) and Aomatsu et al. (2011) used BWT > 3 mm, either alone or in conjunction with presence of color Doppler. Dell’Era et al. (2023) and Khan et al. (2024) employed a uniform BWT ≥ 4 mm cut-off, while Ruess et al. (2000) defined abnormality as BWT ≥ 3 mm and presence of Doppler signal.

Overall heterogeneity amongst studies was substantial: definitions differed in absolute millimeter cut-offs, whether thresholds were uniform or segment-specific, and whether hyperemia or structural changes were obligatory. Pediatric-specific evidence was scant (4 studies).

### Sonographic endpoints and therapeutic response

Thirty-two interventional studies evaluated TMH or TMR as treatment endpoints across various therapeutic classes. TMH was consistently associated with favorable outcomes, including higher rates of corticosteroid-free remission, reduced hospitalization, and decreased treatment escalation, often outperforming mucosal healing as a prognostic marker.^28^^,^^35^^,^^75^ TMR, while a less stringent endpoint, was strongly predictive of subsequent TMH, mucosal healing, and sustained remission, supporting its role as an early treatment-response marker.^28^^,^^35^^,^^36^ Across therapies, anti-TNF agents were most frequently studied (22/32, 68.8%),^3^^,^^6^^,^^8^^,^^17^^,^^19^^,^^23^^,^^25^^,^^27^^,^^30^^,^^37^^,^^43^^,^^47^^,^^48^^,^^76^^,^^77^ followed by ustekinumab,^6^^,^^21^^,^^38^^,^^40^^,^^42^ vedolizumab,^6^^,^^38^ and exclusive enteral nutrition.^78^ Reported TMH rates ranged from 14% to 38% at 12 months, with higher stringency in definitions yielding lower rates but greater prognostic value. Early TMR, particularly at 12-14 weeks, correlated with favorable long-term outcomes across multiple agents (see Supplemental Material for detailed per-study definitions, patient populations, and outcome measures).

### Correlation with clinical disease activity indices

Eighteen studies compared IUS parameters with validated clinical indices, most commonly the HBI and Crohn’s Disease Activity Index (CDAI).^79^ Six studies evaluated correlation between HBI and IUS parameters.^34^^,^^65^^,^^76^^,^^80–82^ While BWT and composite IUS scores such as BUSS and IBUS-SAS showed significant associations with HBI in some cohorts,^34^^,^^65^ another study found no significant difference in HBI between patients with and without IUS-defined active inflammation.^82^ Similar variability was observed for CDAI, with correlations stronger for multi-parameter ultrasound activity indices than for BWT alone,^54^^,^^58^^,^^63^^,^^83^ and correlation ranging from weak (*r* = 0.2)^54^^,^^83^ to moderate (*r* = 0.388)^58^ [see Supplemental Material for further details on per-study coefficients, significance values, and scoring systems used].

### Correlation with other imaging modalities

Eight studies correlated IUS with other cross-sectional imaging modalities. Six studies compared IUS with magnetic resonance imaging (MRI) or magnetic resonance enterography (MRE)^18^^,^^56^^,^^58^^,^^63^^,^^84^^,^^85^ Martínez et al. (2009) reported excellent concordance between IUS and MRI for detecting affected segments (*κ* = 0.905).^63^ The authors concluded that both modalities are complementary, though IUS offered better accessibility and terminal ileum assessment. Similarly, Pascu et al. (2004) found that IUS and MRI correlated comparably with clinical and biochemical markers but noted that IUS added value by assessing peristalsis and vascularity.^58^ Horjus Talabur Horje et al. (2015) demonstrated a strong correlation between IUS and MRE disease activity scores (*r* = 0.84), both aligning well with endoscopic inflammation.^84^ El-Nakeep et al. (2024) observed excellent agreement in bowel wall thickness (ICC = 0.88) and moderate concordance for hyperemia and enhancement.^56^ Castiglione et al. (2017) found near-perfect agreement for detecting TMH (*κ *= 0.90) between IUS and MRE, with both modalities correlating more strongly with mucosal than clinical remission.^18^

Two studies directly compared IUS with computed tomography enterography (CTE) in CD.^56^^,^^75^ In a prospective cohort, Ma et al. (2021) reported strong agreement between IUS and CTE for detecting TMH (*κ* = 0.793; 97% concordance).^75^ Similarly, El-Nakeep et al. (2024) found strong agreement between IUS and CTE for bowel wall thickness (89.7%) and loss of bowel wall echostratification (83.1%) in a cross-sectional study.^56^ One study correlated abdominal X-ray findings with IUS parameters.^51^

### Correlation with endoscopy

Thirty-one studies compared IUS parameters with endoscopic assessment of CD activity activity,^3^^,^^8^^,^^21^^,^^23^^,^^24^^,^^26^^,^^33–35^^,^^38^^,^^42^^,^^44^^,^^51–53^^,^^58^^,^^60^^,^^61^^,^^64^^,^^67^^,^^75^^,^^76^^,^^83^^,^^84^^,^^86–92^ demonstrating moderate-to-excellent agreement despite methodological heterogeneity. Most (16/31 studies) relied on the SES-CD,^3^^,^^21^^,^^23^^,^^24^^,^^26^^,^^34^^,^^35^^,^^42^^,^^44^^,^^75^^,^^76^^,^^84^^,^^86^^,^^91–93^ seven focused on postoperative recurrence using the Rutgeerts score,^53^^,^^61^^,^^83^^,^^87^^,^^88^^,^^90^^,^^94^ and 2 employed the CD Endoscopic Index of Severity.^33^^,^^60^ The remaining studies compared IUS with global endoscopic impressions without a numerical score. BWT and color Doppler parameters showed strong correlations with endoscopic severity (*ρ* or *r*  ≈ 0.55-0.83),^24^^,^^33^ with composite indices such as IBUS-SAS and BUSS performing similarly.^34^^,^^76^ Diagnostic accuracy was highest when BWT thresholds of 3-4 mm were combined with Doppler signal and preserved stratification, yielding AUROC values up to 0.95.^24^ In postoperative monitoring, IUS detected recurrence with sensitivities of 77%-100% but variable specificity, improved by adding CEUS or fecal calprotectin.^87^^,^^90^ A single study in small bowel CD showed moderate agreement between IUS and video capsule endoscopy for therapeutic response^49^ (see Supplemental Material for detailed per-study performance metrics).

### Special populations

Nine studies evaluated pediatric patients with CD or suspected IBD, generally applying adult-derived sonographic thresholds to define abnormality, most commonly BWT > 3 mm.^32^^,^^37^^,^^50^^,^^69–74^ Only 2 studies applied comprehensive TMH definitions,^32^^,^^37^ and one defined TMR.^32^ Across studies, reliance on adult cut-offs was noted as a limitation, underscoring the need for pediatric-specific criteria. Correlations between IUS parameters and the Pediatric Crohn’s Disease Activity Index (PCDAI) were variable, with several studies highlighting discordance between clinical indices and sonographic inflammation.^70^^,^^73^^,^^95^ IUS was also associated with biochemical and endoscopic markers, including fecal calprotectin and SES-CD,^73^ and demonstrated early treatment-response changes more suggestive of TMR than TMH.^70^ One study in pregnant patients reported high accuracy for detecting active disease and strong correlation with fecal calprotectin, though visualization was limited in late gestation^66^ (see Supplemental Material for descriptive full study-level data).

## Discussion

This systematic review identified and synthesized published sonographic definitions of TMH, TMR, and normal or abnormal bowel wall in CD. Across 83 studies, approximately half-defined TMH, one quarter of studies defined TMR, and the remainder provided criteria for normal vs. abnormal bowel. BWT was the dominant parameter in all definitions. TMH was most often defined as BWT ≤ 3 mm, occasionally with segment-specific thresholds up to 4 mm in the rectum, whereas TMR required BWT reduction, typically an absolute decrease of ≥ 1-2 mm or a relative reduction of ≥ 25%. Doppler flow was included in nearly two-thirds of definitions, typically requiring absent or near-absent signal, and fewer incorporated preserved bowel wall echostratification, absence of mesenteric fat wrapping, or resolution of complications.

Despite this substantial body of work, heterogeneity rather than paucity of data is the major barrier to cross-study comparison. TMH definitions ranged from BWT alone to 2 parameter rules (BWT plus Doppler) and multicomponent frameworks adding structural features or complications. TMR definitions similarly varied in whether they required concurrent Doppler improvement or additional structural criteria. Even basic definitions of “normal” bowel wall diverged, with thresholds spanning > 1.5 mm to > 4 mm for BWT and inconsistent requirements for CDS or echostratification. Such variability underscores the need for consensus definitions to generate reproducible, comparable, and ultimately regulatory-acceptable IUS endpoints. The generally moderate risk of bias and incomplete reporting of key methodological domains, such as blinding and interobserver reliability, underscore the need for standardized acquisition and interpretation frameworks in future IUS research.

Recent reviews further highlight this heterogeneity. Allocca et al. (2025) and Sands et al. (2025) synthesized largely adult, treatment-response cohorts, whereas our broader eligibility yielded nearly twice as many CD-specific studies.^2^^,^^12^ Thus, while Allocca et al. provide a treatment-trial perspective across the IBD spectrum, our review provides a broader landscape of structural healing definitions in CD, highlighting areas, particularly pediatric thresholds, and composite-score validation, where consensus is still required.

Guideline statements similarly reflect progress but not consensus. The 2018 European Federation of Societies for Ultrasound in Medicine and Biology (EFSUMB) IUS guidelines recommended a BWT > 3 mm as the diagnostic cut-off (with > 4 mm to maximize specificity) and recognized Doppler and stratification as adjuncts but did not define TMH or TMR.^96^ This absence of standardized definitions was reiterated in the recent ECCO-ESGAR-ESP-IBUS guidelines on diagnostics and monitoring in IBD, which emphasize the need for validated sonographic response and healing criteria.^97^^,^^98^ The 2024 American Gastroenterological Association Clinical Practice Update advanced IUS as a treat-to-target tool, emphasizing BWT and Doppler reduction (≈25%) as markers of response, but again without uniform definitions.^5^ Building on these prior gaps, a 2025 international consensus led by Allocca et al. has now proposed standardized IUS endpoints for clinical trials in IBD, defining ultrasound response as a ≥ 25% reduction in BWT alone or in combination with a ≥ 1-grade reduction in CDS, and remission as normalization of BWT ≤ 3 mm with or without normalization of ancillary parameters. These recommendations mark the first globally harmonized attempt to align IUS-based response and remission criteria across CD and UC, providing a structured framework for future validation in both research and clinical practice. The findings of our systematic review contextualize and support this emerging consensus, highlighting how heterogeneous early definitions have now converged toward the parameters adopted in this framework.

Empirical work illustrates both the challenge and potential of a tiered approach. Helwig et al. analyzed 351 IBD patients in the TRUST programs using 3 hierarchical ultrasound definitions: simplified, extended, and complete TMH.^36^ Healing rates at week 12 ranged from 24%-37%, with greater stringency conferring stronger prognostic value for clinical remission and long-term sonographic improvement. Similarly, while composite indices such as BUSS, IBUS-SAS, and SPAUSS have been used less frequently, they enhance reproducibility, correlate with endoscopic outcomes, and provide quantifiable thresholds that may serve as pragmatic definitions of TMR or TMH, though pediatric validation is lacking.

Pediatric data remain limited and methodologically inconsistent. Our review retrieved 9 studies with pediatric participants with CD. All but one relied on the adult BWT normalized values and only 2 reports specified a comprehensive TMH endpoint.^32^^,^^37^ Guo et al. (2025) was the sole study to define TMR.^32^ No study tested growth-adjusted BWT targets or validated pediatric composite indices. The only dedicated score, SPAUSS, has yet to be externally validated.^69^ These gaps mirror the agenda set by the IBUS Pediatric Committee, which calls for prospective derivation of age-appropriate thresholds, standardized color Doppler grading criteria and harmonized surveillance schedules.^99^ By assembling the full pediatric evidence base, omitted from earlier systematic reviews, our analysis underscores the urgent need for multicenter studies to establish and validate pediatric-specific TMR and healing benchmarks.

Incorporating standardized sonographic definitions into practice has immediate clinical utility. Early IUS assessments, particularly week 12, can identify TMR as a marker of treatment response and TMH as a more stringent prognostic endpoint, enabling timely therapy escalation or de-escalation and reducing reliance on colonoscopy. The 2024 AGA Clinical Practice Update on IUS endorses this strategy suggesting BWT and color Doppler flow change as practical early markers of therapeutic response that predict long-term control.^5^ In light of the growing evidence that specific TMR and TMH thresholds are predictive of sustained clinical and endoscopic outcomes, a formal Delphi consensus, ideally led by the international bowel ultrasound group (IBUS) and IUS group of the United States and Canada (IUSCAN), is now justified to standardize definitions and reporting criteria. This effort would establish the foundation for a unified framework for TMR and TMH.

**Figure 2:**
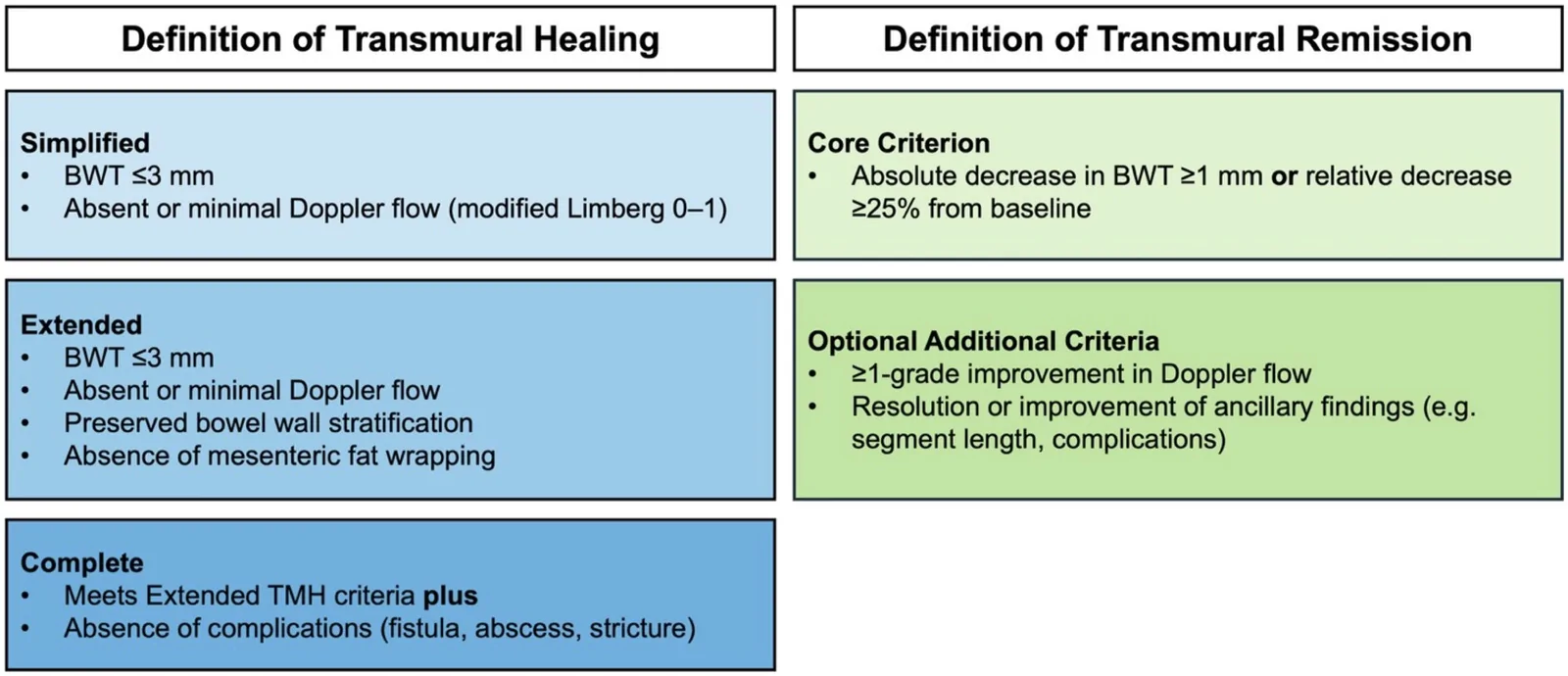
Proposed definitions of TMH and TMR on IUS in CD. TMH definitions are presented as a hierarchical framework with 3 tiers of increasing stringency: Simplified (BWT ≤ 3 mm and absent or minimal Doppler flow), extended (adds preserved bowel wall stratification and absence of mesenteric fat wrapping), and complete (adds absence of complications such as fistula, abscess, or stricture). TMR is defined by an absolute reduction in BWT ≥ 1 mm or relative decrease ≥ 25% from baseline, with optional criteria including Doppler improvement and resolution of ancillary findings.

To address the heterogeneity identified in this review and illustrate a scalable approach applicable to both clinical trials and routine practice, we outline a potential framework for classifying IUS targets (Figure 2). The proposed tiered framework is not intended to define definitive criteria, but rather to reflect common patterns in the literature and highlight feasible targets for prospective validation. A “simplified” TMH construct, limited to the 2 parameters most consistently reported and reproducible across studies (BWT ≤ 3 mm and absent/minimal color Doppler signal [modified Limberg 0-1]), reflects the most common definition in the included literature and aligns with the IBUS Expert Consensus Statement.^11^ For TMR, an absolute (≥1mm) and/or relative (≥25%) BWT reduction, with optional ≥ 1-grade improvement in CDS, appears most consistent with prior consensus proposals.^11^ While not intended as prescriptive criteria, this tiered structure which progresses from simplified to more comprehensive definitions that integrate structural restoration and complication resolution may help harmonize reporting in future research and facilitate meta-analytical synthesis, while remaining feasible for use in routine care.

Our review has several methodological strengths including an exhaustive search spanning 1992-2025, independent, duplicate screening and data extraction to minimize selection error, and formal study appraisal with the JBI risk-of-bias tools, which enabled transparent weighting of evidence. However, important limitations must be acknowledged. Reference standards varied widely, ranging from ileocolonoscopy to biomarkers and other cross-sectional imaging modalities, introducing heterogeneity that precluded quantitative pooling. Another limitation of our review is the lack of description of long-term outcomes utilizing these definitions in the studies reviewed. As most studies were retrospective with heterogeneous populations of varying CD severity, this also limited a robust review of long-term outcomes based on the TMH and TMR definitions. Lastly, several pediatric cohorts were small and only one study assessed IUS in pregnancy, which constrains generalizability. These factors should be considered when interpreting the results.

Future efforts should shift from cataloguing definitions to large-scale prospective validation. The proposed 3-tier TMH/TMR framework should be tested prospectively in multicenter cohorts across different geographic regions and operator expertise to assess its generalizability. Pediatric-specific studies are equally critical; growth-adjusted BWT cut-offs need to be derived and linked to endoscopic and clinical outcomes, rather than extrapolated from adult data. Incorporating IUS targets into adaptive trial designs could allow early TMH/TMR milestones to guide escalation or, when futile, timely de-escalation of therapy. An extension of Strengthening the Reporting of Observational Studies in Epidemiology (STROBE) for IUS is recommended to standardize reporting, alongside development of a core outcome set for transmural endpoints. Long-term cost-effectiveness analyses are warranted to quantify the economic implications of IUS-guided treat-to-target management.

## Conclusion

This systematic review demonstrates that IUS provides a feasible and prognostically meaningful modality for monitoring transmural disease activity in CD; however, its clinical application remains limited by heterogeneity in existing definitions. Our analysis confirms that BWT ≤ 3 mm remains the primary criterion for TMH, while adjunctive color Doppler grading, structural, and composite-score parameters are applied inconsistently, with particularly limited data in pediatric populations. Standardization of ultrasound-based parameters, integrated with endoscopic and biochemical targets, will advance treat-to-target strategies, enable robust cross-study comparisons, and support regulatory recognition of ultrasound as a core outcome measure in future clinical trials.

## Supplementary Material

izag052_Supplementary_Data

## Data Availability

Data and analytic methods will be made available to other researchers upon request.
