# Supplementary material for: Ultrasound criteria for transmural healing and response in Crohn’s disease: a systematic review of definitions and thresholds
Source: Inflamm Bowel Dis. 2026 Jun 1;32(9):1707–21. doi: 10.1093/ibd/izag052 (PMC13533201; doi:10.1093/ibd/izag052)
Supplement: Supplementary file 1 [file izag052_supplementary_data.zip › Supplementary Table I JBI quality assessment.docx]

**Supplementary Table I. Methodological Quality Assessment of Included Studies Using the JBI Critical Appraisal Checklist**

| Author Year | Items on JBI checklist | Total 'yes' of overall total | Yes (n) | No (n) | Unclear (n) | N/A (n) |
| --- | --- | --- | --- | --- | --- | --- |
| Spencer 2023 | 11 | 45% | 5 | 1 | 1 | 4 |
| Cheng 2023 | 11 | 82% | 9 | 2 | 0 | 0 |
| Huang 2023 | 11 | 73% | 8 | 0 | 1 | 2 |
| DeCristofaro 2023 | 11 | 73% | 8 | 1 | 0 | 2 |
| Dell'Era 2023 | 8 | 100% | 8 | 0 | 0 | 0 |
| Nasuno 2023 | 8 | 100% | 8 | 0 | 0 | 0 |
| Furfaro 2023 | 8 | 100% | 8 | 0 | 0 | 0 |
| Zhou 2023 | 8 | 100% | 8 | 0 | 0 | 0 |
| Macedo 2022 | 8 | 100% | 8 | 0 | 0 | 0 |
| Paredes 2022 | 8 | 100% | 8 | 0 | 0 | 0 |
| Ma 2021 | 11 | 73% | 8 | 1 | 0 | 2 |
| Vaughan 2023 | 8 | 88% | 7 | 1 | 0 | 0 |
| Kucharzik 2023 | 11 | 82% | 9 | 0 | 0 | 2 |
| deVoogd 2022 | 11 | 82% | 9 | 0 | 0 | 2 |
| Onali 2022 | 11 | 100% | 11 | 0 | 0 | 0 |
| Smith 2022 | 11 | 100% | 11 | 0 | 0 | 0 |
| Vaughan 2022 | 11 | 91% | 10 | 0 | 1 | 0 |
| Wu 2022 | 8 | 100% | 8 | 0 | 0 | 0 |
| Chen 2022 | 11 | 91% | 10 | 0 | 1 | 0 |
| Schulberg 2022 | 11 | 100% | 11 | 0 | 0 | 0 |
| Allocca 2022 | 11 | 73% | 8 | 0 | 1 | 2 |
| Helwig 2022 | 11 | 73% | 8 | 0 | 1 | 2 |
| Calabrese 2022 | 11 | 82% | 9 | 0 | 0 | 2 |
| Hoffmann 2022 | 11 | 73% | 8 | 0 | 1 | 2 |
| Saevik 2022 | 8 | 100% | 8 | 0 | 0 | 0 |
| Miranda 2021 | 11 | 82% | 9 | 0 | 0 | 2 |
| Marin 2021 | 8 | 75% | 6 | 2 | 0 | 0 |
| Celikyay 2021 | 8 | 63% | 5 | 3 | 0 | 0 |
| Goertz 2021 | 8 | 88% | 7 | 1 | 0 | 0 |
| SuarezFerrer 2021 | 11 | 55% | 6 | 2 | 1 | 2 |
| Dolinger 2021 | 11 | 91% | 10 | 1 | 0 | 0 |
| Flanagan 2020 | 11 | 73% | 8 | 2 | 0 | 1 |
| Zorzi 2020 | 11 | 91% | 10 | 1 | 0 | 0 |
| Kellar 2019 | 11 | 55% | 6 | 1 | 0 | 4 |
| Paredes 2019 | 11 | 82% | 9 | 2 | 0 | 0 |
| Castiglione 2019 | 11 | 73% | 8 | 3 | 0 | 0 |
| Martinez 2019 | 11 | 64% | 7 | 1 | 0 | 3 |
| Wilkens 2018 | 11 | 55% | 6 | 1 | 0 | 4 |
| Orlando 2018 | 11 | 82% | 9 | 2 | 0 | 0 |
| Na 2017 | 11 | 45% | 5 | 2 | 0 | 4 |
| Castiglione 2017 | 11 | 73% | 8 | 3 | 0 | 0 |
| Kucharzik 2017 | 11 | 91% | 10 | 0 | 1 | 0 |
| Chen 2019 | 11 | 82% | 9 | 0 | 2 | 0 |
| Civitelli 2016 | 11 | 82% | 9 | 0 | 2 | 0 |
| Ripolles 2016 | 11 | 82% | 9 | 0 | 2 | 0 |
| Medellin-Kowalewski 2016 | 8 | 75% | 6 | 2 | 0 | 0 |
| HorjusTalaburHorje 2015 | 11 | 55% | 6 | 4 | 0 | 1 |
| Moreno 2014 | 11 | 73% | 8 | 0 | 1 | 2 |
| Castiglione 2013 | 11 | 82% | 9 | 2 | 0 | 0 |
| Cammarota 2013 | 11 | 55% | 6 | 2 | 1 | 2 |
| Paredes 2013 | 8 | 75% | 6 | 2 | 0 | 0 |
| Aomatsu 2011 | 11 | 73% | 8 | 1 | 0 | 2 |
| Onali 2010 | 11 | 64% | 7 | 2 | 2 | 0 |
| Paredes 2010 | 11 | 73% | 8 | 2 | 0 | 1 |
| Calabrese 2009 | 8 | 63% | 5 | 3 | 0 | 0 |
| Martinez 2009 | 8 | 75% | 6 | 2 | 0 | 0 |
| Ripolles 2008 | 11 | 64% | 7 | 4 | 0 | 0 |
| Rispo 2006 | 10 | 70% | 7 | 1 | 2 | 0 |
| Parente 2004 | 10 | 60% | 6 | 1 | 2 | 1 |
| Pascu 2004 | 10 | 70% | 7 | 0 | 2 | 1 |
| Castiglione 2004 | 10 | 90% | 9 | 0 | 0 | 1 |
| Haber 2002 | 10 | 40% | 4 | 4 | 2 | 0 |
| Ruess 2000 | 10 | 40% | 4 | 1 | 3 | 2 |
| Mayer 2000 | 10 | 50% | 5 | 1 | 3 | 1 |
| Hata 1992 | 10 | 40% | 4 | 2 | 3 | 1 |
| Han 2022 | 11 | 82% | 9 | 1 | 0 | 1 |
| Castiglione 2022 | 11 | 64% | 7 | 0 | 0 | 3 |
| Liu 2015 | 8 | 75% | 6 | 0 | 2 | 0 |
| Voogd 2022 | 11 | 91% | 10 | 0 | 1 | 0 |
| Maconi 2001 | 11 | 100% | 11 | 0 | 0 | 0 |
| You 2023 | 11 | 64% | 7 | 1 | 1 | 2 |
| Ukashi 2024 | 11 | 64% | 7 | 1 | 0 | 2 |
| Allocca 2024 | 11 | 64% | 7 | 1 | 0 | 3 |
| Maconi 2024 | 11 | 82% | 9 | 0 | 0 | 2 |
| Wang 2024 | 10 | 60% | 6 | 2 | 2 | 0 |
| Huang 2024 | 11 | 82% | 9 | 0 | 0 | 2 |
| Wu 2025 | 11 | 64% | 7 | 2 | 0 | 2 |
| Guo 2025 | 11 | 73% | 8 | 1 | 0 | 2 |
| Brodersen 2024 | 11 | 64% | 7 | 2 | 0 | 2 |
| El-Nakeep 2024 | 11 | 55% | 6 | 2 | 0 | 3 |
| Yzet 2024 | 8 | 88% | 7 | 1 | 0 | 0 |
| Lorente 2024 | 11 | 64% | 7 | 1 | 0 | 2 |
| Gu 2024 | 10 | 80% | 8 | 2 | 0 | 0 |
| Khan 2024 | 10 | 80% | 8 | 1 | 1 | 0 |
