# Supplementary material for: Ultrasound criteria for transmural healing and response in Crohn’s disease: a systematic review of definitions and thresholds
Source: Inflamm Bowel Dis. 2026 Jun 1;32(9):1707–21. doi: 10.1093/ibd/izag052 (PMC13533201; doi:10.1093/ibd/izag052)
Supplement: Supplementary file 1 [file izag052_supplementary_data.zip › SUPPLEMENTAL MATERIAL.docx]

**SUPPLEMENTAL MATERIAL**

A. **The use of sonographic endpoints to assess therapeutic response**

Thirty-two studies had interventional designs utilizing IUS-based targets. Six studies evaluated TMH after initiating IBD therapies^22,28,34–36,75^. In a large observational cohort, Ma et al. (2021) found that TMH independently predicted steroid-free remission, reduced hospitalization, and reduced the need for treatment escalation, outperforming mucosal healing in multivariate analysis^75^. Smith et al. (2022) similarly reported that TMH at week 12 was linked with higher rates of corticosteroid-free remission at week 48, particularly among patients treated with biologics such as ustekinumab or anti-TNF agents^28^. In a real-world study, Allocca et al. (2022) demonstrated that reductions in ultrasound activity scores were associated with both mucosal and clinical response, while follow-up data from Allocca et al. (2024) confirmed that TMH correlated with sustained remission and decreased treatment intensification^34,35^. Helwig et al. (2022) further showed that early TMH at week 12 significantly increased the odds of clinical remission and favorable sonographic outcomes at one year^36^. Castiglione et al. (2022) explored the concept of deep remission by evaluating the combined achievement of clinical, mucosal, and TMH alongside biochemical normalization^22^. They outlined TMH as a component of deep remission and found that in combined with TMH, patients with fecal calprotectin <94 μg/g and CRP <5 mg/L had the highest likelihood of achieving sustained remission.

Three interventional studies included TMR endpoints^28,35,36^. In a prospective multicenter cohort, Smith et al. (2022) found that patients achieving TMR were significantly more likely to attain corticosteroid-free clinical remission at week 48, with this association remaining independent of mucosal healing status. Similarly, Allocca et al. (2024) found TMR strongly predictive of subsequent TMH, mucosal healing, and sustained clinical remission^28,35^. Patients with early TMR had markedly lower rates of treatment escalation during follow-up. In a single-center prospective cohort, Helwig et al. (2022) reported that TMR at week 12 was associated with increased odds of both short-term clinical remission (OR 3.33–15.2) and long-term sonographic remission at 52 weeks (OR 5.59)^36^.

With regards to evaluating therapeutic response, anti-TNF agents were the most frequently studied (22/32, 68.8%). Sixteen studies evaluated TMH following anti-TNF induction or optimization^3,6,8,17,19,23,25,27,30,37,43,47,48,76,77,81^. In adult cohorts, TMH rates ranged from 25% at one year^17^ to 38.4% in a multicenter study assessing adalimumab-treated patients^43^. Several studies linked TMH with favorable clinical and biochemical outcomes. In a prospective study of infliximab-treated patients, Huang et al. (2024) reported that postinduction BWT <4.5 mm best predicted long-term TMH at 12 months (AUC 0.85), while inflammatory mesenteric fat and elevated IBUS-SAS were negative predictors^76^. Similarly, Calabrese et al. (2022) reported that the proportion of patients achieving TMH increased over time during biologic therapy, from 16% at 3 months to 28% at 12 months, with healing more frequently observed in colonic (48%) than ileal (24%) segments^6^. Two studies explored the relationship between TMH and drug exposure: Han et al. (2022) found that patients with TMR at week 14 had significantly higher median anti-TNF drug levels (e.g., 5.9 µg/mL infliximab vs. 0.9 µg/mL; p < 0.001)^27^, and Lorente et al. (2024) showed a significant correlation between higher adalimumab levels and TMH during the first year of treatment, but not beyond^77^.

Five studies evaluated TMR following anti-TNF therapy, using variable definitions but consistently reporting early sonographic improvement as a meaningful treatment marker^25,27,43,47,48^. Ripollés et al. (2016) demonstrated that over 65% of patients showed sonographic response within two weeks of induction^48^. Sonographic improvement closely aligned with early clinical and biochemical improvement. Similarly, Paredes et al (2010) found that 65% of clinical responders exhibited sonographic response, though normalization of BWT and Doppler occurred in fewer patients (29–47%)^47^. Paredes et al (2019) reported that patients who achieved TMR had reduced rates of treatment escalation and higher rates of sustained remission, highlighting its prognostic utility^25^. Han et al. (2022) found that TMR at week 14 was observed in 47% of patients and strongly correlated with higher infliximab and adalimumab trough levels^27^. Importantly, TMR predicted favorable long-term outcomes, including maintenance of clinical remission. In a more recent study, Maconi et al (2024) reported TMR in 47.1% of patients treated with anti-TNF, and was associated with shorter disease duration, non-stricturing phenotype, and higher anti-TNF drug concentrations^43^.

IUS-based endpoints for ustekinumab, an IL-12/23 antagonist, were evaluated in five studies^6,21,38,40,42^, which reported both TMH and TMR. TMH rates at 12 months ranged from 25% to 29.9%. In a multicenter cohort, Kucharzik et al. (2023) reported TMR in 39.5% and TMH in 25.3% of patients. Both endpoints were associated with clinical remission and biochemical normalization^42^. Similarly, Miranda et al. (2021) observed TMR in 38% and TMH in 26% of patients at one year, with steroid-free remission achieved more frequently among those with TMH^21^. Hoffmann et al. (2022) assessed IUS at week 8 and found that 43% of patients demonstrated a substantial sonographic response, defined as a ≥1 mm reduction in BWT^40^. Among these, 90% had a concurrent ≥70-point decrease in Crohn’s disease activity score (CDAI) and 80% had a CRP reduction ≥0.5 mg/dL, supporting the role of early TMR as a marker of treatment efficacy. Lastly, Calabrese et al. (2022) reported progressive increases in TMH with increasing time treated with ustekinumab, although specific rates for TMR were not delineated^6^.

Two studies evaluated IUS-based endpoints for vedolizumab-treated cohorts^6,38^. Rates of IUS-defined TMH for patients treated with Ustekinumab and vedolizumab were not specifically reported in Onali et al, 2022^38^. Calabrese et al. (2022) showed a consistent decline in IUS disease activity scores associated with increased TMH rates with prolonged treatment^6^.

Only one study examined the impact of exclusive enteral nutrition (EEN) with TMH as an endpoint^78^. Complete mucosal healing (SES-CD ≤1) was achieved in 79% of patients, while only 17% achieved TMH as defined by normalization of bowel sonography parameters including BWT ≤3 mm. Although significant reductions in BWT and complications such as fistulas and abscesses were observed, sonographic signs of transmural inflammation persisted in many patients even after endoscopic healing^78^.

B. **Sonographic endpoints and clinical disease activity indices**

Within the included studies, eighteen studies compared IUS parameters with validated clinical disease activity indices. While many studies reported clinical indices such as HBI, studies that specifically evaluated correlation between HBI and IUS parameters were limited (*n* = 7). In a prospective cohort, Huang et al. (2024) demonstrated that patients who achieved TMH had significantly lower HBI scores throughout follow-up^76^. Allocca et al. (2022) demonstrated a statistically significant correlation between the BUSS and HBI (r = 0.57, p < 0.0001)^34^. You et al. (2023) reported a moderate positive correlation between segmental IUS activity (IBUS-SAS) and HBI (r = 0.62), with increasing HBI scores observed in patients with more extensive disease involvement (median HBI: 1.2 in inactive disease, 2.3 in small bowel disease, and 9.0 in colonic disease)^65^. De Cristofaro et al. (2023) demonstrated a statistically significant inverse correlation between hypoechoic echopattern of bowel (i.e. loss of echostratification) on IUS and HBI (r = –0.208, p = 0.0321), suggesting that changes in sonographic features may reflect inflammation not captured with symptom scores^80^. Cheng et al. (2023) observed that baseline inflammatory mesenteric fat was correlated with less improvement in HBI following treatment with infliximab^81^. In contrast, Celikyay et al. (2021) found no significant difference in HBI between patients with and without IUS-defined active inflammation^82^.

Four studies evaluated the relationship between IUS parameters and the CDAI^54,58,63,83^ Pascu et al. (2004) reported a moderate correlation between an ultrasound activity index developed by the authors which is based on bowel wall thickness, Doppler signal, wall stratification and contrast enhancement, and CDAI (r = 0.388, p = 0.015), stronger than correlations observed with MRI and CDAI^58^. Similarly, Martinez et al. (2009) found that BWT was the IUS parameter most strongly associated with CDAI, although correlation coefficients were not reported^63^. In contrast, Calabrese et al. (2009) reported a weak and non-significant correlation between BWT and CDAI (r = 0.2, p = 0.1)^54,83^. Mayer et al. (2000) reached similar conclusions, with only a weak correlation between CDAI and sonographic inflammation (r = 0.22)^54^.

C. **Sonographic endpoints and correlation with endoscopy**

A total of 31 studies directly compared IUS parameters with endoscopic assessment of CD activity^3,8,21,23,24,26,33–35,38,42,44,51–53,58,60,61,64,67,75,76,83,84,86–92^. Collectively, they confirm a moderate-to-excellent association between transmural sonographic findings and mucosal inflammation, despite methodological heterogeneity. Formal endoscopic scoring systems were used in 19 investigations: 15 relied on the Simple Endoscopic Score for Crohn’s Disease (SES-CD)^3,21,23,24,26,34,35,42,44,67,75,76,84,86,91,92^, seven focused on postoperative recurrence using the Rutgeerts score^53,61,83,87,88,90,94^, and two employed the CD Endoscopic Index of Severity (CDEIS)^33^. The remaining studies compared IUS with global endoscopic impressions without a numerical score.

When expressed as correlation coefficients, key grayscale or color Doppler parameters correlated strongly with endoscopic severity, Spearman’s ρ (or Pearson’s r) ranged from 0.55 to 0.83. Representative examples include BWT versus SES-CD (ρ = 0.75 in Wu 2022) and BWT versus CDEIS (r = 0.67 in Moreno 2014)^24,33^. The composite indices IBUS-SAS and BUSS both showed moderate to strong correlation with SES-CD^34,35,76^.

Diagnostic-accuracy analyses were heterogeneous but converged on pragmatic BWT thresholds of >3–4 mm (occasionally >5 mm) and Limberg/color-Doppler grades ≥ 1 to demarcate active disease. With these cut-offs, sensitivity spanned 56–100% and specificity 61-100%, yielding area-under-the-ROC values as high as 0.95 when BWT was combined with Doppler signal and preserved stratification^24^.

Seven studies evaluated postoperative surveillance after ileocolonic resection^53,61,83,87,88,90,94^. Using the Rutgeerts score as reference, IUS detected endoscopic recurrence with sensitivities of 77-100 %, but specificity was more variable (20-88%), reflecting the difficulty of distinguishing minor i1 lesions from transmural sequelae. Agreement with endoscopy was moderate (range of κ between 0.5-0.67)^61,83,87^ and improved when CEUS or fecal calprotectin (FCP) was added, reducing false-negative rates to <5 % in large cohorts^87,90^.

One prospective study in small bowel CD initiating biologic therapy reported correlation between IUS and video capsule endoscopy (VCE) using the VCE Lewis score at baseline and weeks 14 and 52^49^. The baseline VCE Lewis score correlated strongly with TI BWT (r = 0.65), Limberg Doppler grade (r = 0.60), and the IBUS-SAS (r = 0.65); correlations attenuated over time but remained moderate for the Limberg index (r = 0.47 at follow-up). Agreement between modalities was moderate for therapeutic response (κ = 0.44-0.51) and fair for remission (κ = 0.27).

**D. Special populations**

Nine studies focused on pediatric populations with CD or suspected IBD, with several key themes emerging for definitions of TMR and TMH^32,37,50,69–74^. There was a total of 452 pediatric participants (median 50 [17-74]]. Most pediatric studies adapted adult-derived sonographic thresholds to define abnormality. The majority used a BWT threshold of >3 mm to denote inflammation, though one study^72^ utilized a higher cut-off of ≥4 mm. Comprehensive definitions of TMH were applied in studies by Civitelli et al (2016) and Guo et al (2025), which included BWT ≤3 mm, absence of hyperemia, preserved echostratification, and no evidence of mesenteric fat proliferation or complications, mirroring criteria used in adult cohorts^32,37^. Guo et al (2025) included a definition of TMR, which was defined as a ≥25% or ≥2 mm reduction in BWT. Although the paper by Kellar et al (2019) did not specifically defined TMH or TMR, the authors proposed a novel pediatric-specific score to identify IUS parameters to identify disease activity, the Simple Pediatric Activity Ultrasound Score (SPAUSS), which integrates BWT, color Doppler grade, and mesenteric fat wrapping, though this has yet to be externally validated^69^. Across studies, the reliance on adult cut-offs was noted as a limitation, underscoring the need for pediatric-specific thresholds.

Three studies evaluated the association between IUS findings and the Pediatric Crohn’s Disease Activity Index (PCDAI), or weighted PCDAI (wPCDAI)^70,73,95^. In a prospective study of 33 children, Haber et al (2000) demonstrated that BWT strongly correlated with PCDAI, with higher thresholds (>3 mm) corresponding to active disease based on PCDAI and normalization aligning with remission based on PCDAI^95^. Dolinger et al (2021) demonstrated significant clinical improvement in pediatric patients following infliximab induction, however no significant correlation between wPCDAI and IUS parameters was appreciated^70^.

Several studies demonstrated valuable associations between IUS parameters and other markers of disease activity. In the study by Ruess et al (2000), BWT and color Doppler grades significantly correlated with erythrocyte sedimentation rate (ESR) and multiple clinical indices for both CD and UC^74^. More recent work by Khan et al (2024) reported strong correlations between IUS disease activity scores, endoscopic indices including the Mayo score and SES-CD (r = 0.565 and 0.731, respectively), and fecal calprotectin (r = 0.513)^73^. No significant correlation was observed between IUS disease activity indices and clinical indices such as PUCAI or PCDAI. Dolinger et al (2021) found that bowel wall hyperemia improved earlier than BWT in pediatric patients receiving induction therapy with infliximab and that changes in FCP correlated with reductions in length of bowel that was inflamed on IUS, but not BWT^70^. Similarly, Dell’Era et al (2023) reported that a combination of BWT >3 mm, loss of echostratification, and mesenteric hypertrophy had superior accuracy for diagnosing IBD in comparison to increased BWT alone, with specificity exceeding 90% for each parameter^72^.

Two pediatric studies utilized IUS-defined endpoints to evaluate treatment response. Civitelli et al (2016) assessed TMH following anti-TNF therapy and found that while TMH was rare (14%), partial improvements in BWT and color Doppler grading, more suggestive of TMR, were common^37^. Dolinger et al (2021) evaluated early changes in IUS parameters following infliximab induction therapy and demonstrated significant reductions in color dopper grading, but not BWT, by week 14, again more suggestive of TMR versus TMH^70^. Collectively, these studies illustrate the utility of IUS as a non-invasive monitoring tool in pediatric interventional trials, though variability in outcome definitions limits comparability.

One study evaluated IUS in pregnant patients with IBD. In a prospective cohort of 38 patients followed across all trimesters, De Voogd et al (2022) utilized IUS to assess disease activity and treatment response and compared it to a composite reference standard of clinical indices (i.e., HBI for patients with CD) and FCP^66^. IUS disease activity was defined by BWT thresholds specific to each segment (i.e. >2.0 mm in the TI, >3.0 mm in the colon), alongside increased color Doppler signal, loss of bowel wall stratification, haustration, or mesenteric fat wrapping. TMH was defined as normalization of BWT and absence of any abnormal features. In this population, IUS demonstrated high overall accuracy for identifying active disease (sensitivity 84%, specificity 98%; κ=0.84, p<0.0001), with strong correlation to FCP (r = 0.73) and moderate correlation to clinical indices (r = 0.60). Visualization was adequate for most colonic segments but declined significantly for the TI and sigmoid colon in the third trimester due to uterine enlargement. IUS detected treatment response with 80% sensitivity and 92% specificity when compared to??? supporting its role as a non-invasive monitoring modality during pregnancy, however, there are notable limitations with advancing trimesters and visualization. Notably, over half of patients with active disease on IUS had involvement of multiple segments, and sonographic findings influenced treatment decisions in nearly one-third of encounters.
